# A career reflection

**DOI:** 10.3389/fphys.2025.1678950

**Published:** 2025-09-08

**Authors:** Sandra G. Velleman

**Affiliations:** Department of Animal Sciences, The Ohio State University, Wooster, OH, United States

**Keywords:** chickens, growth selection, heterogeneity, muscle, satellite cells, turkeys

## Introduction

Being asked to write an opinion paper for the Frontiers in Avian Physiology Lifetime Achievements Topic is an honor. While considering this paper and what research area to focus on, I began reflecting on my career as a scientist, which commenced over 40 years ago. I reflected on all the mountains and detours that had to be overcome and the opportunities along the way. With the current challenging times for pursuing scientific research, I decided to provide a career perspective outlining my scientific journey instead of writing an opinion paper on a specific research topic.

My interest in science started as a young girl growing up outside of Boston. When I was 6 years old, I proudly announced to my mother that I was going to be a veterinarian. I had always been drawn to animals and was passionate about biological science. My mother helped fuel these interests by maintaining a membership at the Museum of Science in Boston. Every Saturday, I would attend science classes followed by a visit to their library to select books for us to read together during the week. I still remember the instructor’s name and some of the demonstrations carried out to illustrate scientific concepts. The time spent at the Museum of Science helped build my scientific passion. In school, I was placed in the honors science curriculum and took 10th-grade biology in the ninth grade. I was so fortunate to have a teacher who recognized my research skills. One day, she told me I was not going to be a veterinarian but a research scientist. I did not even know what a research scientist did, and to be honest, I was crushed. She signed my yearbook, saying, “My budding scientist.” At that time, I thought I would prove her wrong by becoming a veterinarian. She saw something in me that I did not, and I am forever grateful to her for planting the seed of a research scientist in my mind.

My undergraduate studies could be summarized as follows: I obtained my BA degree with distinction in Biology from Boston University in 1981. This statement would severely underrepresent this transformative period of my life. I was fortunate to take Cell Biology with a new assistant professor, Dr. Robert E. Hausman. I loved the Cell Biology course and was excited to attend lectures and learn as much as possible. One day, I finally mustered the courage to attend Dr. Hausman’s office hours and ask if I could switch advisors to him. The meeting changed my life. In addition to him becoming my advisor, he asked if I would like to work in his laboratory during the summer to help set it up. At the end of the summer, Dr. Hausman asked if I would like to apply for the research honors program. As in ninth grade, someone saw something in me that I did not recognize. My undergraduate research was on prostaglandin E1 during embryonic muscle development, resulting in my first peer-reviewed research publication in Biophysical Biochemical Research Communication in 1981 (Hausman and Velleman, 1981). More importantly, my lifelong interest in the cellular communication mechanisms that lead to tissue and organ formation was formed. Opportunities often present themselves as minor steps in your life but can result in a new life direction. I am not sure where my life would be if I had not gone to Dr. Hausman’s office hours. My life certainly would have been different, and that is all I know.

To further pursue cellular communication, my interest evolved into the new area of how the extrinsic environment outside the cell, the extracellular matrix, affected cellular behavior. I pursued my doctoral studies with Dr. Paul F. Goetinck at the University of Connecticut, focusing on the role of the extracellular matrix environment in avian limb development. After completing my doctorate in 1986, I was eager to learn emerging molecular biology techniques to study human diseases involving the extracellular matrix. To achieve this goal, I was accepted as a National Institutes of Health postdoctoral trainee at the University of Pennsylvania Medical School’s Connective Tissue Research Institute. During my doctoral studies, I had thought that I wanted to leave academia and pursue osteoarthritis research in industry. Gaining molecular biology skills was essential. However, I realized that working with humans, especially children presenting with various connective tissue disorders, was very different from working with research animals. It troubled me to witness children with severe connective tissue disorders, such as osteogenesis imperfecta. After much deliberation, I realized I did not have the emotional makeup for the career I had thought would be my future direction and needed to find a new path. Another opportunity then presented itself—a hidden, life-changing pathway for me. At the University of Connecticut, an NIH program project grant to the Health Center, and they needed someone to lead the animal component of the research. It was explained to me with much chagrin that I would be in the Animal Science Department, which was a part of the College of Agricultural Sciences. My previous research had used an avian model, but I was always part of a biology department, not one focused on domestic animal research. I accepted the postdoctoral position, which included the option to develop my own research program for the future.

After 2 years in the postdoctoral position at the University of Connecticut, I was promoted to a non-tenure-track assistant professor of Animal Science, focused on developing a new research program in avian muscle growth mechanisms. At a biotechnology meeting held at the University of Connecticut, I met Dr. Douglas McFarland from South Dakota State University, who presented his research on avian breast muscle adult myoblasts, or satellite cells. These are the cells responsible for the regeneration and growth of muscle. I asked Dr. McFarland if he thought satellite cells could be regulated by the extracellular matrix. At that time, the extracellular matrix had only been identified in connective tissue, and muscle was not a connective tissue. My instinct was that the extracellular matrix might be an important component in non-connective tissues. I took a big risk, but Dr. McFarland was excited about collaborating with me on this. Hence, another life-changing opportunity arose through attending a meeting and asking a question. In 1998, I published our research results demonstrating that avian breast muscle satellite cells produce their own extracellular matrix (Velleman, 1998). This was a groundbreaking finding that completely challenged the current dogma. We now know that the extracellular matrix is present in all tissues and organs, regulating biological homeostasis and growth, maintaining structural organization, and supporting regeneration. My message here is to hold onto your beliefs, even if established findings oppose them; you may be contributing significant new knowledge.

In 1995, I transitioned into a tenure-track assistant professorship in the Department of Animal Sciences at The Ohio State University. I was promoted through the ranks, and in 2022, I received the honorific title of Distinguished Professor of Food, Agricultural, and Environmental Sciences in Animal Sciences. In 2024, I retired and became a professor emeritus in the Department of Animal Sciences. During my tenure at both the University of Connecticut and The Ohio State University, the greatest joy has been impacting the lives of those I trained. My goal was to provide opportunities to others, similar to those that made such a huge difference in my life trajectory. My undergraduate and graduate students and postdoctoral researchers represent my legacy.

During my career, I published over 215 peer reviewed research publications, 8 book chapters, and have co-edited 8 books. My career was accompanied with disappointments too. You must stay strong during these periods of doubt to achieve your goals. Of my discoveries, the following are the ones which I deem most important.

## Satellite cells produce their own extracellular matrix extrinsic environment

The extracellular matrix includes collagens, proteoglycans, and non-collagenous glycoproteins and was believed to be produced by only connective tissue cells that are important in the structural support of tissues such as bone and cartilage. Non-connective tissues, such as muscle, were not thought to produce extracellular matrix proteins. In 1998 (Velleman, 1998), my laboratory was the first to show that breast muscle satellite cells synthesized extracellular matrix heparan sulfate proteoglycans. Heparan sulfate proteoglycans are a group of extracellular matrix macromolecules linking muscle cells to their extrinsic environment and regulating growth through fibroblast growth factor 2 signal transduction. This was one of the first demonstrations of an extracellular matrix protein being synthesized by a non-connective-tissue cell type and having a function beyond just structural support. Subsequent studies through the years focused on two families of heparan sulfate proteoglycans, syndecans and glypicans, and how they differentially regulate breast muscle cell proliferation and differentiation.

## Heterogeneity of satellite cells

Muscle growth in poultry occurs through the formation of myofibers or hyperplasia of myoblasts, with myoblasts fusing to form multinucleated myotubes that mature into muscle fibers and muscle fiber bundles. After hatching, all muscle growth is from satellite cells donating their nuclei to existing muscle fibers, resulting in the enlargement of myofibers through hypertrophy. Although commonly thought of as a homogenous single-cell population, satellite cells are composed of multiple populations with differing proliferation and differentiation in poultry. The research we conducted has shown that growth selection in poultry has altered the types of satellite cells present in the breast muscle and their biological activity (Velleman, 2022; Xu et al., 2023; Xu and Velleman, 2023). In broilers, satellite cell proliferation and differentiation have decreased with growth selection, whereas the opposite has occurred in meat-type turkeys (Xu and Velleman, 2023). Decreased satellite cell proliferation and differentiation, especially in broilers, may be associated with muscle fiber degenerative myopathies such as wooden breast. The satellite cell is the cell type responsible for the repair and regeneration of myofibers, which is likely suppressed with decreased satellite cell biological activity.

## Effects of post-hatch extrinsic conditions (temperature and nutrition) on muscle development and meat quality

Feed restrictions are used by the industry, and newly hatched chicks and poults can be exposed to hot and cold temperatures during handling and transportation. In addition to a potential to reduce the final breast muscle weight, these extrinsic stimuli immediately after hatching can also change the expression of muscle-specific genes and cause the conversion of the satellite cells to an adipogenic cell fate, increasing fat within the breast muscle (Velleman et al., 2010; Velleman et al., 2014a; Velleman et al., 2014b). Xu et al. (2022) showed that proliferating satellite cells are more sensitive to temperature than differentiating satellite cells in the expression of adipogenic genes. Immediately after hatching, satellite cells rapidly proliferate, increasing their responsiveness. Selection for growth in turkeys has increased the thermal sensitivity of satellite cells. When satellite cells exhibit their highest mitotic activity immediately after hatching, with increased proliferation, they are capable of expressing adipogenic genes, which may significantly affect the composition and morphological structure of the breast muscle. A key finding from my research is that administering feed restrictions during the first week post-hatch leads to increased intramuscular lipid accumulation in the market-age breast muscle. However, if a feed restriction is applied during the second week post-hatch, after the period of maximal satellite cell activity, increased intramuscular fat accumulation is eliminated in the market-age breast muscle.

## Maternal inheritance of breast muscle morphology in turkeys

Commercial turkeys are produced by crossing a sire line usually selected for greater muscling and growth. During studies of muscle development in several line crosses, it was observed that the breast muscle morphology of the offspring crosses was always the same as that of the female parent around market age (Velleman and Nestor, 2004). These findings indicated maternal inheritance of breast muscle morphological structure. This was one of the first observations of maternal inheritance influencing a trait of economic importance in a domestic animal.

The most appropriate way to summarize my career is one of others recognizing talent in me and providing opportunities. For those early in their career, always remain open to new paths. A new path might not be presented as a dramatic modification but perhaps as something subtle in nature. As a professional, I have tried to make a difference in the lives of my students by presenting them with new chances and adding novel information to the knowledge database. In the future, I hope others will continue pursue satellite cell heterogeneity coupled with differential expression of extracellular matrix genes as this is a key element in both the growth of muscle and onset of myopathies.
